# Validation of the hip disability osteoarthritis outcome Score-12 shortform in a German cohort

**DOI:** 10.1007/s00402-024-05561-6

**Published:** 2024-09-26

**Authors:** Matthias Büttner, Felix Wunderlich, Philipp Drees, Yama Afghanyar, Sebastian Fischer, Philipp Schippers, Lukas Eckhard

**Affiliations:** 1 Institute of Medical Biostatistics, Epidemiology and Informatics (IMBEI), University Medical Center of the Johannes Gutenberg University Mainz, Mainz, Germany; 2 Department of Orthopaedics and Traumatology, University Medical Center of the Johannes- Gutenberg University Mainz, Langenbeckstrasse 1, 55131 Mainz, Germany; 3 Department of Foot and Ankle Surgery, Berufsgenossenschaftliche Unfallklinik Frankfurt am Main, 60389 Frankfurt, Germany; 4 Mainz, Germany

**Keywords:** HOOS-12, Validation, Osteoarthritis, Patient reported outcome measures, PROMs, THA

## Abstract

**Introduction:**

To consider the inherent respondent burden of PROMs, the HOOS-12 as a shortform of the well-established HOOS questionnaire has been published. While the HOOS-12 has been validated in an initial study, further evaluation in other, non-English speaking cohorts is necessary. We therefore aimed to evaluate responsiveness, convergent construct validity, internal consistency, and floor and ceiling effects of the HOOS-12 in a cross-sectoral german cohort.

**Materials and Methods:**

European Quality of Life 5 Dimensions scores and HOOS data of patients undergoing total hip arthroplasty (THA) were used for the analyses. HOOS-12 scores were calculated from the full length HOOS. Statistical analysis was conducted, investigating internal consistency, floor and ceiling effects, convergent and discriminant validity, responsiveness, and known-group comparisons.

**Results:**

A ceiling effect was present for postoperative HOOS-12 score and its pain, function and QoL subscales. Internal consistency was high between baseline and all follow ups, inter-item correlation was high (Cronbach’s alpha > 0.30) for HOOS-12 score and all subscales. Correlation of HOOS-12 pain with HOOS pain was high (*r* = 0.9). Correlation of HOOS-12 function with HOOS ADL and HOOS S/R was high (*r* = 0.89, *r* = 0.74). Correlation was moderate between HOOS-12 pain and HOOS-12 function with its respective EQ-5D score (*r*=-0.58, *r*=-0.59).

**Conclusion:**

The HOOS-12 showed good convergent construct validity and is responsive to changes in pain, function, QoL and hip impact between preoperatively and 1 year postoperatively. A substantial ceiling effect for all subscales at 1 year postoperatively limits the ability to capture variance across particularly well performing patients.

**Trial Registration:**

The Trial is registered with the German Clinical Trials Register (https://www.drks.de; DRKS00013972; 23 March 23, 2018).

## Introduction

Patient reported outcome measures (PROMs) are frequently used to evaluate postoperative outcome from a patient’s perspective and provide information on pain, function and quality of life for informed clinical decision-making. In order to provide a more patient-centred assessment of surgical outcomes after total joint arthroplasty, PROMs have been included in arthroplasty registries and value-based reimbursement systems [1, 2].

In the selection of PROMs for the routine use in clinical care and registries, respondent burden needs to be considered. The HOOS-12 is a recently published shortform of the widely used Hip dysfunction and osteoarthritis Outcome Score (HOOS) [3, 4]. It comprises of 12 questions from the original 40-item HOOS and provides Pain, Function and Quality of Life (QoL) scales as well as a summary hip impact score.

While the psychometric properties of the HOOS-12 have been investigated by its originators in an initial validation study, it was noted that further evaluation of its performance in other joint replacement cohorts is necessary. Furthermore, to date the HOOS-12 has not been studied in a non-English speaking cohort. We therefore aimed to evaluate responsiveness, convergent construct validity, internal consistency, and floor and ceiling effects of the HOOS-12 in a cross-sectoral German cohort.

## Materials and methods

### Study design

HOOS-12 data of patients who received total hip arthroplasty (THA) as part of the cross-sectoral, multi-center, prospective PROMISE trial was used for the analyses in this investigation. The PROMISE study protocol has been previously published [6]. In short, patients scheduled for THA for the treatment of osteoarthritis in one of three hospitals covering different care sectors (a regional hospital, an orthopedic-specialized hospital, and a tertiary referral academic hospital) were enrolled. Exclusion criteria were life expectancy less than 1 year (e.g., advanced cancer), any conditions that might preclude elective surgical intervention, and medical or psychological factors that would prevent patients from participating or providing informed written consent. Treatment was performed using standardized pathways following enhance recovery after surgery (ERAS) principles.

### Data collection

Baseline demographic data included age, sex, comorbidities and diagnosis. A validated German translation of the HOOS [6–8] and the European Quality of Life 5 Dimensions (EQ-5D, 5 level version) scores were collected at the baseline preoperative outpatient clinic, 3 months (± 2 weeks), 6 months (± 2 weeks), and 12 months (± 2 weeks) after surgery using electronic case report forms. HOOS-12 subscale scores and the summary impact score were calculated from the full length HOOS according to the method described by its originators [4]. The HOOS score evaluates symptoms and function in patients suffering from hip osteoarthritis and patients undergoing total hip replacement. It contains of items from the following five subscales, partly derived from the “Western Ontario and McMaster Universities Osteoarthritis Index” (WOMAC): “symptoms”, “pain”, “activity of daily living” (ADL), “sports/recreation” (S/R) and “quality of life”. Items are scored on a 5-point Likert scale. Scores range from 0 to 4, with 0 representing the worst stage. A single overall score is generated, ranging from 0 to 48, with higher scores indicating better results The survey was handed to included patients after informed consent during their visit at the outpatient clinic.

The EQ-5D is a five item measure of health-related quality of life that covers mobility, personal care, usual activities, pain/discomfort, and anxiety/depression [9]. 12 months post diagnosis, participants additionally filled out a questionnaire which measured if in certain domains (e.g. pain) their levels are back at normality or have at least improved. Patients could select between the following answering options: (1) back at normality or complete improvement, (2) not back at normality but large improvement, (3) not back at normality but moderate improvement and (4) not back at normality but little improvement.

### Ethics, registration and funding

The PROMISE trial was conducted in accordance with the latest versions of the Declaration of Helsinki, Good Epidemiological Practice, and local regulatory requirements, including the German Federal Data Protection Act (Bundesdatenschutzgesetz). The protocol was approved by the ethics committees of the participating federal states: Rhineland-Palatinate [837.533.17 (11367)], Baden-Wuerttemberg [B-F-2018-042], and Hesse [MC 84/2018]. The protocol is registered with the German Clinical Trials Register (DRKS00013972). Written informed consent was obtained from all patients before enrolment. The PROMISE trial was supported by a grant from the Federal Joint Committee (01NVF16015).

### Statistical analysis

Scores for the HOOS, HOOS-12 and EQ-5D-5 L were computed to the published scoring guidelines. Sample characteristics are given as means or percentages according to the data. Internal consistency for the HOOS-12 scales was assessed by calculating Cronbach’s alpha and the inter-item correlation for the respective scales. Floor and ceiling effects are given as percentages, describing the share of participants with the highest or lowest possible score at the respective time points. For assessing convergent validity, Spearman correlations were calculated for the HOOS-12 scales and the matching HOOS and EQ-5D-5L scales. Discriminant validity was determined by calculating Spearman correlations for the HOOS-12 scales and the anxiety/depression scale of the EQ-5D-5L. In order to assess the responsiveness of the German version of the HOOS-12, effect sizes and standardized response means between baseline and the later time point was calculated assuming that patients functionality improves over time. Additionally, an anchor based approach was chosen to establish responsiveness. It was checked whether statistically significant differences between the mean scores in the improvement categories from baseline to 12 months post baseline were present. For the HOOS-12 scales the following anchor based questions were used: (1) “pain relief during day time” for the HOOS-12 pain scale, (2) “ability to perform daily household activities” for the HOOS-12 functioning scale, and (3) “ability to participate in social or leisure time activities” for the HOOS-12 QoL scale. Questions were answered on a 4-point scale, ranging from “back to normal, comprehensive improvement” to “not back to normal, slight improvement”. For the summary score of the HOOS-12 no matching anchor question was available. Known group comparisons were performed by dividing the participants into two groups, namely into participants with and without walking aids. Mean comparisons for those two groups were performed using Mann-Whitney-U tests.

All statistical analysis were performed using R (Version 4.1.2; The R-Foundation).

## Results

### Cohort characteristics

935 patients who underwent THA between May 2018 and March 2020 were included in the study (413 male, 522 female; mean age 66.0 (SD 10.6); mean BMI 28.3 kg/m^2^ (SD 5.5)). Table 1 summarizes the cohorts’ baseline demographic and clinical characteristics.

**Table 1:** Cohort baseline data

	Mean (SD) or *n* (%)	Range (if applicable)
**Age**	66.0 (10.6)	23–93
**Sex**		
male	413 (44.2)	
female	522 (55.8)	
**Side operated**		
left	456 (48.8)	
right	453 (48.4)	
both	26 (2.8)	
**ASA**	2.2 (0.6)	
**BMI (m²/kg)**	28.3 (5.5)	15.9–62.4

### Internal consistency

All three HOOS-12 subscales, as well as the summary score demonstrated high Cronbach’s alpha values at baseline, 3-month, 6-month and 12-month follow up indicating internal consistency reliability (Table 2). Good correlation between each item and the respective domain was indicated by inter-item correlation scores exceeding 0.30 for all HOOS-12 subscales as well as the summary score.

**Table 2:** Internal consistency as demonstrated by Cronbach‘s alpha

	baseline	3-months	6-months	12-months
	alpha [95%CI]	alpha [95%CI]	alpha [95%CI]	alpha [95%CI]
HOOS-12 Pain	0.71 [0.68;0.74]	0.83 [0.82;0.85]	0.84 [0.83;0.86]	0.88 [0.87;0.89]
HOOS-12 Function	0.77 [0.75;0.79]	0.84 [0.82;0.86]	0.87 [0.85;0.88]	0.90 [0.88;0.91]
HOOS-12 QOL	0.76 [0.74;0.78]	0.85 [0.85;0.87]	0.86 [0.84;0.88]	0.87 [0.85;0.88]
HOOS-12 Summary	0.88 [0.87;0.89]	0.92 [0.92;0.93]	0.93 [0.92;0.94]	0.94 [0.93;0.95]
	**Inter-Item-Correlation**			
	baseline	3-months	6-months	12-months
HOOS-12 Pain	0.38	0.59	0.61	0.67
HOOS-12 Function	0.46	0.57	0.63	0.69
HOOS-12 QOL	0.45	0.62	0.65	0.67
HOOS-12 Summary	0.38	0.53	0.56	0.61

### Construct validity

Tests of construct validity supported convergent and discriminant validity of HOOS-12 at baseline and one year postop. The correlation of HOOS-12 Pain and HOOS Pain was very high (Baseline: *r* = 0.90; One year postop: *r* = 0.97), indicating that all reliable variance in the full-length HOOS Pain scale was captured by the HOOS-12 Pain scale. The HOOS-12 Function scale also had high correlations with HOOS ADL (Baseline: *r* = 0.89; One year postop: *r* = 0.95) and HOOS S/R (Baseline: *r* = 0.74; One year postop: *r* = 0.87). Moderate to high correlation was found between HOOS-12 Pain and EQ-5D Pain (Baseline: *r*=-0.58; One year postop: *r*=-0.72) and HOOS-12 Function and EQ-5D ADL (Baseline: *r*=-0.59; One year postop: *r*=-0.68). Discriminant validity for HOOS-12 Scales and the fear Item of Eq. 5D-5 L showed low correlation as shown in Table 3.

**Table 3:** Discriminant validity HOOS-12 scales and Eq. 5D-5 L fear

	HOOS-12 Pain		HOOS-12 Function		HOOS-12 QOL	
	baseline	12-months	baseline	12-months	baseline	12-months
Equation 5D-5 L Fear	*r*= -0.19	*r*=-0.37	*r*=-0.22	*r*=-0.38	*r*=-0.31	*r*=-0.41

### Known groups validity

Known groups validity was assessed at baseline grouping patients by whether or not they were dependent on walking aids preoperatively. All HOOS-12 domain and summary scores demonstrated statistically significant differences between these two groups, supporting known groups validity (Table 4).

**Table 4:** Known groups validity at baseline

	Walking aid dependent (*n* = 154)	Non walking aid dependent (*n* = 774)	*P*-value
	Mean (SD)	Mean (SD)	
HOOS-12 Pain	33.3 (19.0)	43.2 (16.3)	< 0.001
HOOS-12 Function	30.1 (17.3)	44.5 (17.3)	< 0.001
HOOS-12 QOL	17.7 (14.8)	27.9 (16.2)	< 0.001
HOOS-12 Summary	27.2 (13.7)	38.6 (14.4)	< 0.001

### Responsiveness

All HOOS-12 subscales and the summary score were responsive to change after THA at all timepoints (3-, 6-, and 12-months post operative), studied as demonstrated by large effect sizes (1.8 to 3.0) and standardized response means (Table 5).

**Table 5:** Responsiveness of HOOS-12 subscales and HOOS-12 summary score

	Baseline vs. 3-months			Baseline vs. 6-months			Baseline vs. 12-months			Effect Size			Standardized Response Mean			
	Baseline	3-months	difference	Baseline	6-months	difference	Baseline	12-months	difference	3-months	6-months	12 months	3-months	6 months	12-months	
HOOS-12 Pain	41.8 (17.2)	82.4 (19.2)	40.6 (23.3)	42.1 (17.1)	86.2 (16.8)	44.0 (21.1)	42.2 (16.6)	89.7 (16.3)	47.5 (20.0)	2.2 [2.1;2.4]	2.6 [2.4;2.8]	2.9 [2.7;3.1]	1,7	2,1	2,4	
HOOS-12 Function	42.3 (17.9)	78.3 (18.3)	35.9 (22.3)	42.7 (17.9)	82.4 (18.0)	39.8 (21.1)	43.1 (17.6)	86.6 (17.3)	43.5 (21.0)	2.0 [1.8;2.1]	2.2 [2.1;2.4]	2.5 [2.3;2.7]	1,6	1,9	2,1	
HOOS-12 QOL	26.4 (16.3)	65.3 (23.7)	39.1 (25.4)	26.8 (16.4)	72.2 (22.8)	45.5 (24.4)	27.0 (16.2)	77.8 (21.8)	50.8 (24.2)	1.9 [1.8;2.0]	2.3 [2.1;2.5]	2.7 [2.5;2.9]	1,5	1,9	2,1	
HOOS-12 Summary	36.9 (14.9)	75.4 (18.4)	38.5 (20.8)	37.3 (14.8)	80.4 (17.3)	43.2 (19.3)	37.5 (14.5)	84.8 (16.8)	47.5 (18.9)	2.3 [2.1;2.5]	2.7 [2.5;2.9]	3.0 [2.8;3.3]	1,8	2,2	2,5	

All values indicated as “mean”; SD is shown in parenthesis; 95%CI is shown in square brackets

### Anchor based approach

Analysis with anchor questions showed significant differences regarding mean scores (baseline to 12- months) in all four groups. Mean differences and p-values are shown in Table 6.

**Table 6:** Mean differences between anchor questions and respective HOOS-12 scores

		Back to normal, comprehensive improvement	Not back to normal, but great improvement	Not back to normal, but moderate improvement	Not back to normal, but slight improvement	
		Mean difference (SD)	Mean difference (SD)	Mean difference (SD)	Mean difference (SD)	p-value
**Pain**						
HOOS- 12 Pain		53.0 (16.5)	40.8 (18.7)	25.3 (17.9)	4.8 (18.4)	< 0.001
HOOS Pain		48.0 (17.0)	37.9 (17.5)	24.6 (17.4)	4.4 (18.1)	< 0.001
**Function**						
HOOS − 12 Function		48.9 (17.8)	40.7 (20.7)	28.0 (18.1)	7.0 (30.2)	< 0.001
HOOS ADL		44.2 (17.5)	37.2 (18.5)	23.9 (16.7)	6.7 (26.4)	< 0.001
HOOS Sport		60.5 (23.9)	53.1 (21.3)	32.9 (26.9)	17.7 (14.4)	< 0.001
**HOOS/HOOS − 12 QOL**		59.1 (20.6)	45.7 (24.2)	24.9 (23.4)	20.8 (18.5)	< 0.001

### Floor and ceiling effects

Floor effects (percentage with the lowest (worst) possible score) for all measures were very low (< 2%) pre- and post-THA. Ceiling effects (percentage with the highest (best) possible score) were negligible at baseline. 12 months post-THA, there were notable ceiling effects for all subscales (Pain 52.8%; Function 40.3%; QoL 23.2%) as well as the summary scale (15.9%; Table 7).

**Table 7:** Floor and ceiling effects of HOOS-12 scores

	% at Floor				% at Ceiling			
	baseline	3 months	6 months	12 months	baseline	3 months	6 months	12 months
HOOS-12 Pain	1,3	0	0	0	5,3	31	39,8	52,8
HOOS-12 Function	1,4	0	0,2	0,2	0,2	16,3	27,6	40,3
HOOS-12 QOL	8,5	0,5	0,1	0,1	0	11,5	18,4	23,2
HOOS-12 Summary	0,4	0	0	0	0	6,4	11,9	15,9

## Discussion

In this analysis of its psychometric properties, the HOOS-12 score for the assessment of outcomes after total hip arthroplasty for osteoarthritis demonstrated internal consistency reliability, good convergent and discriminant validity, known groups validity and no floor effect but a considerable ceiling effect in all its subscales at one year postop. For the first time, the HOOS-12 was assessed in a german speaking cohort and at one year postoperatively outside the initial validation study published by its developers.

In generally well-performing cohorts, detection of change over time and differences between study groups at the upper level of PROMs can be challenging. If a PROM demonstrates an inability to discriminate between good and very good results, variance in outcome is insufficiently measured above a certain level [10]. The ceiling effect is the statistical construct that describes the accumulation of best possible outcomes. This study identified a ceiling effect to be present for all HOOS-12 subscales, as well as the summary score at 1-year postoperatively. Post-operative ceiling effects were previously reported in the initial publication introducing the HOOS-12 by Gandek et al. and in the French validation of Putman et al. [4, 5, 11]. Similarly, Ackerman et al. also found a significant ceiling effect for the Pain (46%), Function (39%) an QoL (26%) subscales as well as the summary score (17%) of the HOOS-12 [12]. Regarding the function subscale, the developers have noted, that questions were selected in order to choose the items most relevant for the majority of patients. Therefore, questions covering more complicated tasks, such as running, squatting or pivoting on the leg originally included in the full-length HOOS were not included in the HOOS-12, which makes the occurrence of a ceiling effect more likely [4]. When studying younger, more active cohorts, it may consequently be beneficial to add scores covering higher-level activities, such as the HOOS-physical function (PS) score. The “pain” subscale exhibited a ceiling effect as well. As it has previously been noted, the construct of pain is inherently limited to a maximum reduction down to “no pain” and is therefore particularly prone to a ceiling effect, regardless of the questions used to assess it [13].

Correlation of the HOOS-12 pain subscale with the HOOS pain subscale, as well as correlation of the HOOS-12 function subscale with the HOOS ADL and S/R subscales were high in this study, indicating that the items chosen for the shortform score sufficiently capture the variance within the cohort studied. This is in line with previous findings [4]. All four QoL questions of the HOOS are included in the HOOS-12, therefore correlation was not assessed.

The HOOS-12 summary score and the subscales were highly responsive to change at one year postoperatively. These findings are similar to the results of Ackerman et al., who found the HOOS-12 to be highly responsive at 6 months postoperatively [12]. Previously, the internal consistency reliability was reported with Cronbach’s alpha ranging from 0.81 to 0.93 in Ackerman et al.’s to 0.77–0.93 in Gandek et al.‘s publication [3, 4, 12]. With Cronbach’s alpha ranging from 0.88 to 0.94, we found slightly higher values, with the summary score showing the highest reliability.

Construct validity had previously been tested evaluating the HOOS-12 in relation to the SF-36 Health Survey, a general measure of physical and mental functioning and well-being [14], and the EQ-5D-5L utility score. Finding only moderate correlations between the HOOS-12 and the EQ-5D in this analysis, the present study confirms previous results, which is explained by relating a hip specific measurement with measures of general health.

This study is not without limitations. Patients completed the full-length HOOS and HOOS-12 scores were calculated from these results rather than using the HOOS-12 as a stand-alone measure. However, as described by the originators, this is a valid way to obtain HOOS-12 results. The results found in this study can only be used for THA-cohorts and should not be generalized to other hip pathologies that might be investigated with the HOOS-12.

The use of a homogenous cohort consisting only of patients undergoing THA for the most relevant diagnosis of osteoarthritis was one of the strengths of this study. At the same time, only patients with a life expectancy below one year and conditions precluding from elective surgery were excluded, leading to a cohort representing the whole of typical arthroplasty patients. In contrast to previous publications, the completion of the HOOS-12 was not optional, and the completion rate was > 95%, eliminating a possible source of bias. In addition, using 1-year postoperative follow-up data, this study provides information about the most relevant timepoint following THA, since to date external validation was only performed for the 6-month timepoint. While the most substantial improvement after total joint replacements is seen within the first 6 months, most studies use the 12-month period to assess functional outcome.

## Conclusions

The HOOS-12 showed good convergent construct validity and is responsive to changes in pain, function, QoL and hip impact between preoperatively and 1 year postoperatively. While it does not exhibit a floor effect, the substantial ceiling effect for all subscales at 1 year postoperatively limits the ability to capture variance across particularly well-performing patients.

## Data Availability

The datasets used and/or analysed during the current study are available from the corresponding author on reasonable request.
